# Quantitative Analysis of Carbon Flow into Photosynthetic Products Functioning as Carbon Storage in the Marine Coccolithophore, *Emiliania huxleyi*

**DOI:** 10.1007/s10126-015-9632-1

**Published:** 2015-04-15

**Authors:** Yoshinori Tsuji, Masatoshi Yamazaki, Iwane Suzuki, Yoshihiro Shiraiwa

**Affiliations:** Faculty of Life and Environmental Sciences, University of Tsukuba, 1-1-1 Tennodai, Tsukuba, Ibaraki 305-8577 Japan; Core Research for Evolutional Science and Technology (CREST), Japan Science and Technology Agency (JST), Tsukuba, 305-8572 Japan; Graduate School of Life and Environmental Sciences, University of Tsukuba, Tsukuba, 305-8572 Japan

**Keywords:** Alkenones, Carbon partitioning, Carbon storage compound, *Emiliania huxleyi*, Haptophyte, Lipid biosynthesis

## Abstract

The bloom-forming coccolithophore *Emiliania huxleyi* (Haptophyta) is a dominant marine phytoplankton, cells of which are covered with calcareous plates (coccoliths). *E. huxleyi* produces unique lipids of C_37_–C_40_ long-chain ketones (alkenones) with two to four *trans*-unsaturated bonds, β-glucan (but not α-glucan) and acid polysaccharide (AP) associated with the morphogenesis of CaCO_3_ crystals in coccoliths. Despite such unique features, there is no detailed information on the patterns of carbon allocation into these compounds. Therefore, we performed quantitative estimation of carbon flow into various macromolecular products by conducting ^14^C-radiotracer experiments using NaH^14^CO_3_ as a substrate. Photosynthetic ^14^C incorporation into low molecular-mass compounds (LMC), extracellular AP, alkenones, and total lipids except alkenones was estimated to be 35, 13, 17, and 25 % of total ^14^C fixation in logarithmic growth phase cells and 33, 19, 18, and 18 % in stationary growth phase cells, respectively. However, less than 1 % of ^14^C was incorporated into β-glucan in both cells. ^14^C-mannitol occupied ca. 5 % of total fixed ^14^C as the most dominant LMC product. Levels of all ^14^C compounds decreased in the dark. Therefore, alkenones and LMC (including mannitol), but not β-glucan, function in carbon/energy storage in *E. huxleyi*, irrespective of the growth phase. Compared with other algae, the low carbon flux into β-glucan is a unique feature of carbon metabolism in *E. huxelyi*.

**Electronic supplementary material:**

The online version of this article (doi:10.1007/s10126-015-9632-1) contains supplementary material, which is available to authorized users.

## Introduction

Coccolithophores play an important role in global oceanic carbon cycling due to their worldwide distribution and capacity to produce huge blooms (Tyrrell and Merico 2004; Harada et al. 2012; Read et al. 2013). The cells are usually covered with calcareous scales, called coccoliths, consisting of CaCO_3_ crystals and acid polysaccharides (AP) called coccolith polysaccharides. Coccolithophores belong to the Haptophyta, which evolved through the secondary endosymbiosis of red algae into a non-photosynthetic eukaryotic host. During this process, many genes were transferred from the endosymbiont to the host cell. Consequently, secondary algae including coccolithophores contain mosaic genomes consisting of genes from endosymbionts and host non-photosynthetic protists (Read et al. 2013). According to recent genomic studies, the newly acquired genes from host protists contribute to the carbon metabolism of secondary algae. For example, the common coccolithophore, *Emiliania huxleyi*, possesses a chloroplast-localized pyruvate carboxylase (PYC) (Tsuji et al. 2012) and an enzyme set corresponding to the ornithine–urea cycle (Allen et al. 2011). In addition, the carbon metabolism of *E. huxleyi* is distinct from that of primary endosymbiotic algae as it yields unique photosynthetic products, such as long-chain unsaturated ketones known as alkenones, water-soluble β-glucan, and acid polysaccharides (Rontani et al. 2006; Vårum et al. 1986; Fichtinger-Schepman et al. 1981).

Alkenones are structurally unique lipids characterized by the presence of extremely long-chain carbon compounds (C_37_–C_40_) with two to four *trans*-double bonds and a *keto*-group in each molecule (Rontani et al. 2006). Several physiological functions of alkenones have been proposed, namely, as membrane components (Sawada and Shiraiwa 2004), buoyancy regulators (Fernández et al. 1996), and storage compounds (Epstein et al. 2001; Prahl et al. 2003; Eltgroth et al. 2005; Pan and Sun 2011). Recently, alkenones were proposed to be a storage compound in *E. huxleyi* since they accumulate in cytosolic lipid droplets under illumination, and their levels decreased under dark conditions, although no comparative evaluation with other photosynthetic products is available (Epstein et al. 2001; Prahl et al. 2003; Eltgroth et al. 2005; Pan and Sun 2011).

Unlike plants and green algae that accumulate water-insoluble α-glucan (starch), *E. huxleyi* produces water-soluble β-glucan, which was assumed to be a storage compound (Vårum et al. 1986). A molecule of β-glucan of *E. huxleyi* contains β-(1 → 6) and β-(1 → 3) linkages and is characterized by a relatively high ratio of β-(1 → 6) linkages (Vårum et al. 1986). In addition to the neutral polysaccharide β-glucan, *E. huxleyi* also produces another type of polysaccharide, AP, known as coccolith polysaccharide, which regulates the morphogenesis of coccoliths by controlling CaCO_3_ crystal growth (Fichtinger-Schepman et al. 1981). AP in *E. huxleyi* consists of mannose polymer as the main chain and side chains with galacturonic acid, xylose, and rhamnose with sulfate groups (Fichtinger-Schepman et al. 1981). AP is synthesized in the intracellular coccolith vesicle, embedded in the CaCO_3_ crystals, and then excreted onto the cell surface with the coccoliths (Van Emburg et al. 1986). Unlike β-glucan, AP is considered to be a structural component of coccoliths rather than an energy storage compound (Van Emburg et al. 1986; Kayano et al. 2011).

In addition to the macromolecules described above, some low molecular-mass compounds (LMC) appear to have an important role in energy storage in *E. huxleyi*. Recently, Obata et al. (2013) showed high carbon flux into mannitol compared with other sugars and amino acids and suggested that mannitol is a potential candidate storage compound.

*E. huxleyi* accumulates unique photosynthetic products and some, such as alkenones and β-glucan, are assumed to be metabolically active energy storage compounds. However, due to the complexity of the fate of fixed carbon, no quantitative analysis of carbon flux into these compounds in *E. huxleyi* has been performed. Previous studies on the synthesis of alkenones and polysaccharides were carried out independently; no quantitative data for simultaneous estimation of carbon flux into alkenones and polysaccharides in the same experiment are available. Consequently, the significance of alkenones and β-glucan as energy storage carbon compounds has not been demonstrated experimentally. This was due to the lack of a useful analytical method for fractionation of carbon storage compounds in marine microalgae, including *E. huxleyi*. In this study, we established the first analytical method to identify major carbon storage compounds in *E. huxleyi*.

## Materials and Methods

### Organism and Culture Conditions

The organism used in this study was the coccolithophore *E. huxleyi* NIES 837 (Haptophyta), which was isolated from the Great Barrier Reef in 1990. The algal cells in the stock culture have been maintained autotrophically in natural seawater enriched with Erd–Schreiber’s medium (NA-ESM), and later the soil extract component of ESM was replaced with 10 nM (final concentration) sodium selenite (modified NS-ESM). We previously found that selenite is an essential micronutrient for *E. huxleyi* growth, and soil extract can be replaced by 10 nM sodium selenite (Danbara and Shiraiwa 1999). The algal cells (50 ml in suspension) were maintained in a 100-ml Erlenmeyer flask under illumination by a 20-W fluorescent lamp at an intensity of 20–30 μmol m^−2^ s^−1^ with a light/dark regime of 16 h/8 h.

In the experimental culture, *E. huxleyi* cells were grown in medium containing the artificial seawater Marine Art SF (produced by Tomita Seiyaku Co., Ltd., Tokushima, Japan and distributed by Osaka Yakken Co. Ltd., Osaka, Japan) enriched with modified ESM (MA-ESM). The composition of Marine Art SF1 was described previously (Danbara and Shiraiwa 1999). Prior to the experiments, cells were pre-cultured for 10–14 days (ca. 5–7 generations) under experimental conditions with various dilutions. Pre-cultured cells were then inoculated to fresh medium following culture to start the experimental culture. For experimental culture, algal suspension (500 ml) in a 1-l Erlenmeyer flask with an air-permeable and bacteria-free porous silicone cap was illuminated continuously by a 20-W fluorescent lamp at an intensity of 120 μmol m^−2^ s^−1^ at 20 °C. The culture was shaken by hand once per day. The pH was maintained at 8.2 by the 10-mM Tris–HCl included in the modified MA-ESM medium.

### Conditions for ^14^C-Labeling Experiments

For the experimental culture, a portion of culture suspension (100 ml) was transferred to another culture bottle (200-ml Erlenmeyer flask) at the logarithmic (2 days after inoculation, 1.5–2.0 × 10^6^ cells ml^−1^) and stationary (8 days after inoculation; 8–12 × 10^6^ cells ml^−1^) growth phases. The ^14^C-labeling experiment was immediately started by injection of 100 μl of NaH^14^CO_3_ (3.7 MBq, 20 μM). The final concentration of dissolved inorganic carbons (DIC) was ca. 2 mM, which is the air-equilibrated level. In the light/dark transition experiments, the light was turned off and the culture bottle was immediately wrapped with aluminum foil. To analyze the time course of ^14^C-labeling, 20 ml of algal suspension were harvested by centrifugation (4400×*g* for 5 min at 4 °C) and washed with 1 ml of fresh modified MA-ESM medium for further analysis of ^14^C-labeled metabolites.

### Fractionation of β-glucan, AP, Alkenones, and Other Compounds

To focus on ^14^C-labeling patterns of putative macromolecular carbon storage compounds, we established a method of separating cellular components into six fractions: (1) low molecular-mass compounds/proteins/nucleic acids (LMC/proteins/NA), (2) external acid polysaccharides located in the extracellular coccoliths (AP*ext*), (3) internal acid polysaccharides presumably located in the coccolith vesicles (AP*int*), (4) β-glucan as a neutral polysaccharide, (5) long-chain ketones (alkenones), and (6) lipids other than alkenones.

**Fig. 1:**
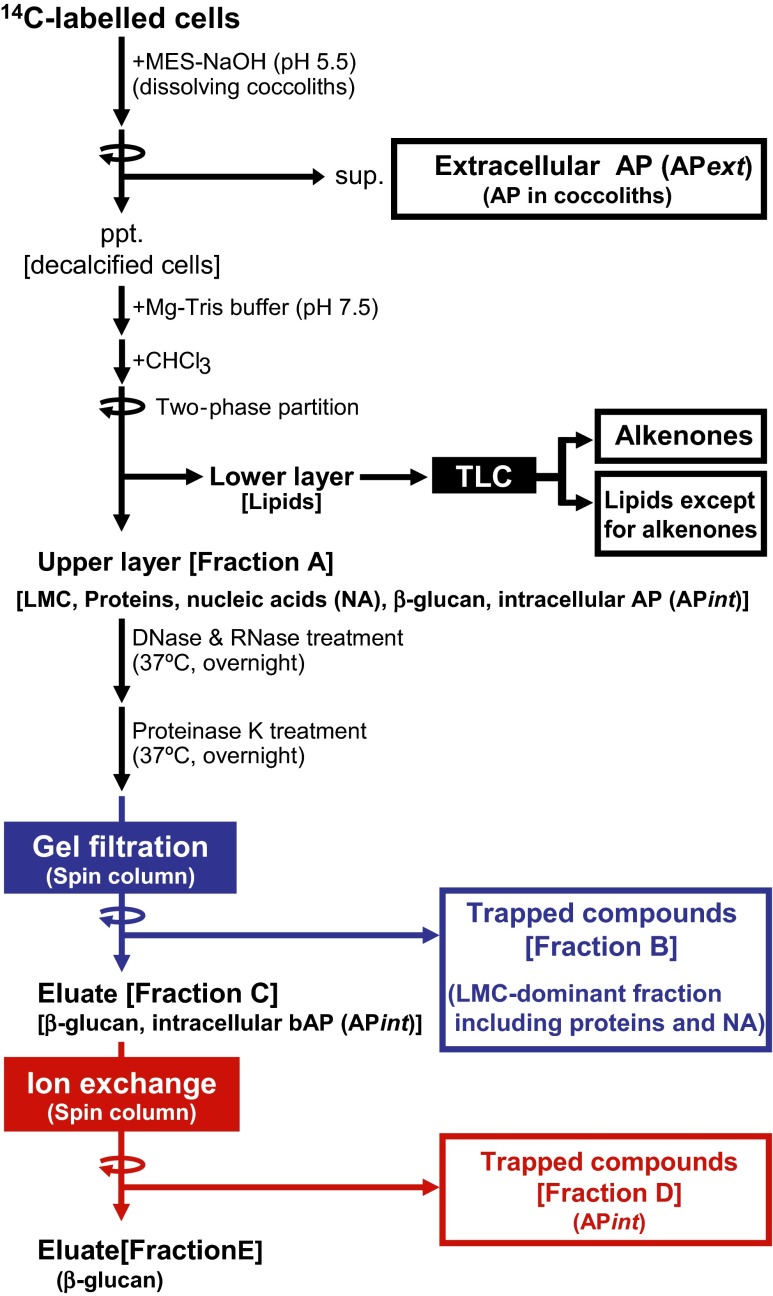
The novel fractionation protocol established in this study. For details, see the “Materials and Methods”

The fractionation procedure is outlined in Fig. 1. Harvested cells were washed with fresh medium as described above. The resultant cell pellets were washed with 1 ml of a decalcifying solution containing 3 % NaCl and 50 mM MES-NaOH (pH 5.5), and then treated again with 0.5 ml of the decalcifying solution to extract AP from the coccoliths (AP*ext*) by dissolving the CaCO_3_ crystals. After combining two supernatant fractions containing AP*ext*, the acidic fraction was exposed to an air stream overnight to remove inorganic ^14^C derived from Ca^14^CO_3_. After the removal of ^14^C-inorganic carbon, ^14^C radioactivity in AP*ext* was quantified using a liquid scintillation counter (LSC) (LSC-6100, Hitachi Aloka Medical, Tokyo, Japan). In the protocol of this study, ^14^C in CaCO_3_ was removed as ^14^CO_2_ by acid treatment, since we focused on fate of photosynthetically fixed carbons. Therefore, ^14^C incorporation into coccoliths was not determined in this study.

The resultant pellets containing artificially decalcified cells were mixed with Mg–Tris buffer (40 mM Tris–HCl, 8.5 mM MgCl_2_, pH 7.5) and chloroform, then separated by centrifugation into aqueous (upper part) and non-aqueous (lower part) fractions by the two-phase partitioning method: a water-soluble fraction (fraction A containing LMC/proteins/NA; Fig. 1) and a chloroform-soluble fraction (lipids). The lipid fraction was subjected to thin-layer chromatography (TLC) and developed by using hexane/ethyl acetate (9:1) as the solvent. On the autoradiogram of the TLC plate, two major ^14^C-labeled bands were detected (bands I and II in Fig. S1a).

To perform the analysis, we prepared a non-radioactive lipid fraction in parallel with the radioactive lipid fraction, since radioactive samples cannot be used in the gas chromatography (GC) system. Non-radioactive lipids and ^14^C-labled lipids were developed in adjacent lanes on TLC, then respective bands in the non-radioactive sample were marked according to the position of the radioactive bands in the adjacent lane. The marked silica gel was extracted by mixtures of hexane/ethyl acetate (95:5), hexane/ethyl acetate (9:1), and MeOH/ethyl acetate (1:1) in sequence. All extracts were then combined and concentrated by evaporation. The concentrated samples were subjected to GC using a flame ionization detector (FID) (GC-2014 AFsc, Shimadzu Seisakusho Co., Kyoto, Japan). Based on GC-FID analysis, bands I and II were identified as C_38_ and C_37_ alkenones, respectively (Fig. S1b, detailed information on the GC-FID analysis is described below). C_39_ alkenones were not detected in the two major bands, possibly due to the low C_39_ alkenone production by strain NIES837 grown at 20 °C, as reported previously (Ono et al. 2009). Therefore, the sum of ^14^C radioactivity in both C_37_ and C_38_ alkenones is expressed as the “alkenone fraction” in this paper.

The upper layer (fraction A) was expected to contain LMCs, proteins, NA, β-glucan, and AP*int*. Fraction A was heated at 70 °C for 10 min to remove contaminating chloroform and obtain the “water-soluble fraction.” Then, deoxyribonuclease (10 units ml^−1^, final concentration) and ribonuclease (50 μg ml^−1^, final concentration) were added and the mixture was incubated at 37 °C overnight to degrade nucleic acids (NA) to nucleotides. Proteinase K (50 μg ml^−1^, final concentration) was then added, and the mixture was incubated at 37 °C overnight to degrade proteins to small peptides.

Next, 150 μl of the enzyme-treated samples were applied to a spin column containing Sephadex G75 gel and centrifuged in a swinging-bucket rotor at 8×*g* for 10 min. Polysaccharides, including β-glucan and AP*int* (fraction C), were present in the eluate, while LMC, nucleotides, and peptides (fraction B) were trapped in the gel (Fig. 1).

The eluate (100 μl) from the Sephadex G75 gel filtration column was applied to a spin column containing DEAE-cellulose and centrifuged at 8×*g* for 10 min in a swinging-bucket rotor. The column was then washed with 100 μl of deionized water to elute compounds not bound completely. The combined eluate fraction contained neutral polysaccharides, namely β-glucan (fraction E), while the fraction retained in the gel contained acid polysaccharides, such as AP*int* (fraction D).

### Preparation of a Standard Sample of Alkenones

We prepared standard alkenones (shown as alkenones extract in supplemental Fig. S1) from *E. huxleyi* cells according to Sawada et al. (1996) with minor modifications. Briefly, cell pellets were extracted using 5 ml of MeOH with sonication, and further extracted using 5 ml of MeOH/dichloromethane (1:1) and 5 ml of dichloromethane. All extracts were combined, and *n*-triacontane (1 mg ml^−1^ at final concentration) was added as an internal standard. After the addition of water (25 ml) and saturated NaCl solution (5 ml), extracts were mixed vigorously. Lipids in the CH_2_Cl_2_ layer were passed through a column containing anhydrous Na_2_SO_4_ to remove water and then dried using an evaporator. Lipids were dissolved in 2 ml of hexane and applied onto a silica gel column. Alkenones were eluted sequentially with hexane, hexane/ethyl acetate (95/5, *v*/*v*) and hexane/ethyl acetate (9:1, *v*/*v*). All eluates were combined and evaporated, and then dissolved using small amounts of hexane for GC-FID analysis. The standard alkenones were also analyzed using GC-mass spectrometry according to the method of Nakamura et al. (2014) to identify peaks of alkenones on the GC chromatogram.

### Analysis of Alkenones by GC-FID

Bands of alkenones extracted from TLC and standard alkenones were analyzed using GC (GC-2014 AFsc, Shimadzu Seisakusho Co., Kyoto, Japan) equipped with FID attached to a capillary column (length, 50 m; internal diameter, 0.32 mm; CP-Sil5 CB; Agilent Technologies Inc., Santa Clara, CA). Helium was used as a carrier at a constant flow rate of 1.25 ml min^−1^ in split-less mode. Temperature was programmed as follows: 60 °C for 1.5 min, an increase to 130 °C at 20 °C min^−1^, a further increase to 300 °C at 4 °C min^−1^ and holding at 300 °C for 25 min.

### Evaluation of Fractionation Efficiency of Water-Soluble Macromolecules

We evaluated the fractionation efficiency of water-soluble macromolecules (included in fraction A, Fig. 1; Table S1) using commercial salmon sperm DNA and self-prepared RNA, AP, β-glucan, and ^35^S-labeled crude proteins as test samples. Since simultaneous quantification of these compounds in the same mixture was difficult, we performed independent assays for each compound.

To evaluate the fractionation efficiency of DNA into fractions B and C (Fig. 1; Table S1), salmon sperm DNA was used as a test sample. Salmon sperm DNA dissolved in Mg–Tris buffer, corresponding to fraction A, was subjected to enzymatic treatment and subsequent gel filtration (Fig. 1). After gel filtration, the DNA content of the eluate was determined using a NanoDrop 1000 (Thermo Scientific, Wilmington, DE, USA). The fractionation efficiency was calculated by comparison of the DNA content of the initial sample and that after gel filtration. To evaluate RNA fractionation efficiency into fractions B and C, total RNA purified from *E. huxleyi* cells was used as the test sample.

To evaluate protein fractionation efficiency, we prepared ^35^S-labeled proteins from *E. huxleyi* cells. For this purpose, 50 ml of *E. huxleyi* cells were labeled with ^35^S-Met/Cys (24 kBq ml^−1^ final concentration) for 24 h under continuous illumination. After labeling, cells were harvested and subjected to two-phase partition with Mg–Tris buffer and chloroform. The upper layer (corresponding to fraction A) was fractionated into fractions B and C by enzymatic treatment and gel filtration. The ^35^S-protein fractionation efficiency was calculated by measuring radioactivity in the samples before and after gel filtration.

To evaluate the fractionation efficiency of AP and β-glucan into fractions B and C, we purified AP and β-glucan from *E. huxleyi* according to Kayano and Shiraiwa (2009). AP purified from whole cell contains AP*int* and AP*ext*, and both APs are structurally the same with different localization patterns. Purified AP was dissolved in Mg–Tris buffer, which corresponds to fraction A, and then subjected to enzymatic treatment and subsequent gel filtration. The AP content of the samples before and after gel filtration was measured by phenol-H_2_SO_4_ assay (Kayano and Shiraiwa 2009). The β-glucan fractionation efficiency was determined using the method described except that AP was replaced by β-glucan.

To determine the AP fractionation efficiency into fractions D and E, purified AP was dissolved in deionized water and then this sample, corresponding to fraction C, was applied to the ion exchange spin column. AP contents of the initial sample and eluate were measured using phenol-H_2_SO_4_ to calculate the fractionation efficiency. Fractionation efficiency of β-glucan into fractions D and E was determined in the same manner, except that AP was replaced by β-glucan.

Proteins, DNA, and RNA in fraction A were recovered at 95, 97, and 98 % from fraction B. Removal rates of proteins and nucleic acids from the polysaccharide fraction (Ffraction C in Fig. 1) were greater than 95 % (Table S1). Neutral and acid polysaccharides, such as β-glucan and AP*int*, respectively, in fraction C were recovered at 100 and 89 % from fractions E and D, respectively (Table S1). The total recovery rates of β-glucan and AP*int* in fraction A were 76 and 87 % from fractions E and D, respectively (Table S1).

### TLC Analysis of LMC

Cell suspension (100 ml) was labeled using NaH^14^CO_3_ as a substrate under continuous illumination. After the 24-h labeling period, cells were harvested by centrifugation and suspended in 500 μl of Mg–Tris buffer. After the addition of 500 μl of chloroform, samples were mixed vigorously and centrifuged briefly to separate the water-soluble compounds from lipids by two-phase partition. One-hundred microliters of the aqueous (upper) layer were transferred to another tube and mixed with 400 μl of methanol. Water-soluble LMC was obtained as the supernatant after precipitation of water-soluble macromolecules by centrifugation at 18,000×*g* for 30 min. The resultant 80 % methanol-soluble fraction was subjected to TLC analysis as the ^14^C-LMC fraction. To identify the ^14^C-mannitol spot on the TLC plate, standard mannitol (0.2 μmol, purchased from Wako Purechemical industries Ltd., Osaka, Japan; Cat. No. 133-00845) was mixed with ^14^C-LMC samples (ca. 3000 dpm), and then developed on TLC according to Tsuji et al. (2009). The addition of standard mannitol was essential since the amount of mannitol contained in the ^14^C-LMC fraction was too low to detect colorimetrically. Autoradiogram of the TLC plate was obtained using Bio-imaging Analyzer System (BAS-1800; Fuji Photo Film, Tokyo, Japan). The mannitol spot was colorimetrically visualized by spraying 0.5 % (*w*/*v*) KMnO_4_ dissolved in 0.1 M NaOH (Bansal et al. 2008).

## Results

### Growth and Photosynthetic ^14^C Fixation in *E. huxleyi*

The main culture of *E. huxleyi* NIES837 was maintained under continuous illumination at a saturated light intensity (120 μmol photons m^−2^ s^−1^) without bubbling. To allow for gas exchange, 500 ml of cell suspension was cultured in a 1-l Erlenmeyer flask with a gas-permeable cap. Medium contained 10 mM Tris and the initial pH of the culture was set at 8.2. Therefore, the concentration of DIC is expected to be at near air-equilibrium levels (ca. 2 mM). Artificial seawater (Marine Art SF-1) enriched with modified Erd–Schreiber’s medium (modified MA-ESM, Danbara and Shiraiwa 1999), which contains 1.9 mM NO_3_^−^ and 28 μM inorganic phosphate (Pi), was used. Since light intensity given during photosynthetic ^14^C-fixation assay was saturated, nutrient limitation, such as inorganic phosphate and nitrate, can be considered to be major factor affecting carbon allocation pattern in LP and SP cells.

**Fig. 2:**
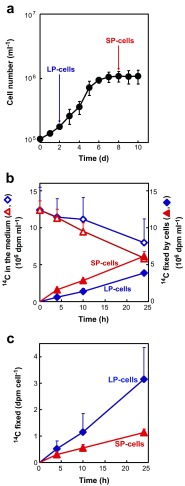
Growth curve of the main batch culture without bubbling or shaking and the time course of photosynthetic ^14^C fixation in the coccolithophorid *Emiliania huxleyi* NIES837. **a** Algal growth curve of the main culture. The culture (500 ml) was maintained under continuous illumination at 120 μmol photons m^−2^ s^−2^. At the logarithmic (LP cells) and stationary (SP cells) growth phases, an aliquot (100 ml) of the culture was transferred to a fresh subculture flask for ^14^C labeling. **b** Time courses of changes in ^14^C remaining in the medium (*open symbols*) and ^14^C incorporated into cells (closed symbols) in LP (*diamonds*) and SP cells (*triangles*). **c** Time courses of ^14^C fixation (calculated as dpm per cell) for LP cells (*closed diamonds*) and SP cells (*closed triangles*). The photosynthetic rates were 0.13 and 0.05 dpm cell^−1^ h^−1^ in LP and SP cells, respectively. Values are means ± SD of three independent experiments. For details of ^14^C-labeling experiments, see the “Materials and Methods.” The temperature was maintained at 20 °C during both culture (**a**) and ^14^C-labeling (**b**, **c**)

Aliquots (100 ml) of cells were withdrawn from the main culture at the logarithmic and stationary growth phases to prepare LP and SP cells, respectively (Fig. 2a). Cells were immediately transferred to other subculture glass vessels for photosynthetic ^14^C-labeling experiments under continuous illumination (Fig. 2b, c). Conditions for labeling experiments were identical those in the main culture, except that 100 ml of cell suspensions was cultured in a 200-ml Erlenmeyer flask. To start the ^14^C-labeling experiments, NaH^14^CO_3_ was injected as a substrate into the vessels. Total ^14^C-incorporation proceeded linearly for 24 h in both LP and SP cells, although ^14^C-bicarbonate concentration in the medium decreased to ca. 50 % of the initial value (Fig. 2b). The rate of ^14^C-fixation (dpm cell^−1^ h^−1^) calculated on a cell basis was 2.7-fold higher in LP than SP cells, showing that LP cells are more photosynthetically active than SP cells (Fig. 2c). While SP cells still had some activity of carbon fixation (Fig. 2c), cell division of SP cells was arrested probably due to the limitation of nutrients (Fig. 2a).

### Establishment of a Fractionation Method for Quantitative Estimation of ^14^C Incorporation into Major Photosynthetic Products in *E. huxleyi*

We developed a fractionation protocol for macromolecules as major photosynthetic products in *E. huxleyi* cells (Fig. 1). According to this method, ^14^C products were fractionated into the following six portions: (1) AP*ext* which is embedded in or surrounding the coccoliths displayed on the cell surface, (2) the LMC-dominant fraction which contains mainly primary photosynthetic metabolites and mannitol together with minor contamination by proteins, nucleic acids (NA), AP*int* and β-glucan (see Supplementary Table S1), (3) AP*int* which is located in intracellular coccolith-producing vesicles, (4) β-glucan, which is neutral polysaccharide, (5) alkenones which are neutral lipids, and (6) lipids except alkenones (Fig. 1).

In this method, water-soluble macromolecules (Fraction A, Fig. 1) were separated into fractions B to E. We evaluated the fractionation efficiency of compounds using commercial salmon sperm DNA (purchased from Sigma-zAldrich Chemical, St. Louis, MO; Cat. No. D1626) and self-prepared RNA, AP, β-glucan and ^35^S-labeled crude proteins as standard test samples (Supplementary Table S1; see the “Materials and Methods” for details). During preparation of fractions B and C, more than 95 % of DNA, RNA, and proteins were present in fractions A and B, while 87 and 85 % of β-glucan and AP, respectively, were recovered in fraction C (Table S1).

The content of ^14^C-LMC in fraction B was independently estimated by omitting enzymatic degradation of proteins and NA (see the Materials and Methods; Fig. S2). Following 24 h ^14^C-labeling of SP cells, ^14^C-LMC comprised ca. 75 % of ^14^C in fraction B; the remainder (25 %) comprised the sum of ^14^C-proteins, ^14^C-NA, and ^14^C-polysaccharides (Fig. S2). According to the fractionation efficiencies shown in Table S1, ^14^C-proteins (95 %), ^14^C-DNA (97 %), ^14^C-RNA (98 %), ^14^C-β-glucan (13 %), and ^14^C-AP*int* (15 %) (% of the total ^14^C in each component) were estimated to be fractionated from fraction A into fraction B.

### Trends in ^14^C Incorporation into Major Photosynthetic Products

**Fig. 3:**
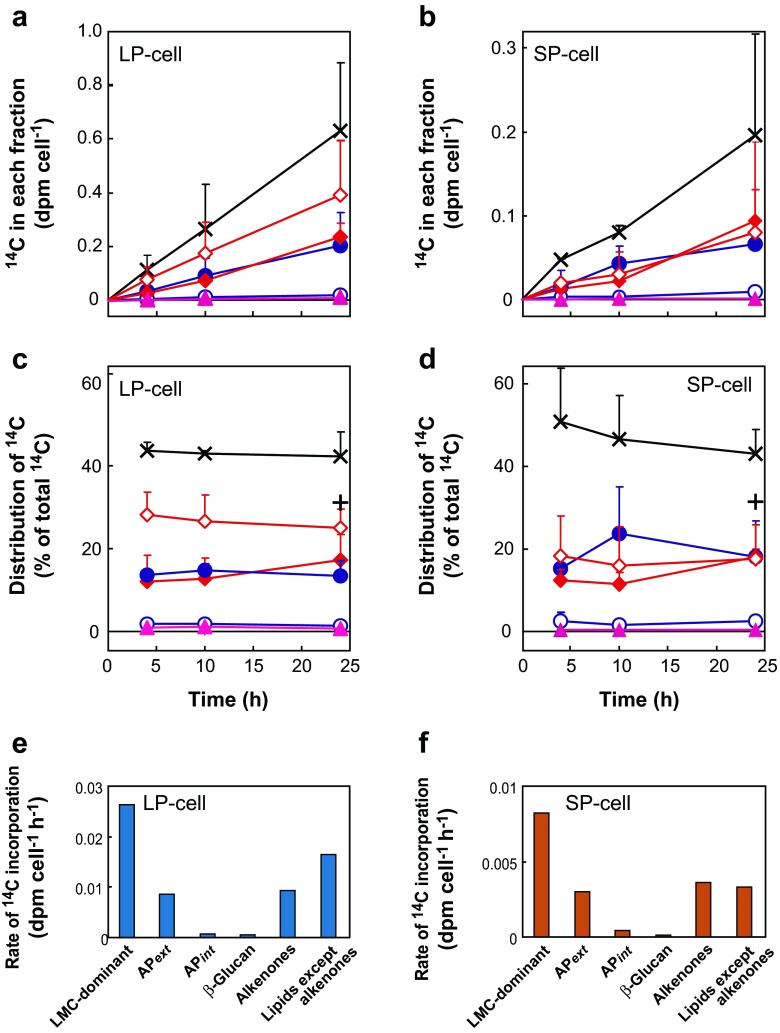
Photosynthetic ^14^C incorporation into various fractions of LP (**a**, **c**, **e**) and SP cells (**b**, **d**, **f**) of the coccolithophore *E. huxleyi* NIES 837. **a**, **b** ^14^C incorporation (dpm cell^−1^) into various fractions. **c**, **d** ^14^C distribution (% of total ^14^C fixation) into each fraction. *Crosses* LMC-dominant fraction including ^14^C-proteins and ^14^C-NA as minor components; *plus sign* ^14^C-LMC value calculated from data obtained separately, as shown in Supplementary Fig. S2 (ca. 75 % of ^14^C in LMC-dominant fraction comprised ^14^C-LMC after 24 h ^14^C-labeling.); *closed circles* extracellular acid polysaccharides (AP*ext*); *open circles* intracellular acid polysaccharides (AP*int*); *closed diamonds* alkenones; *open diamonds* lipids other than alkenones; *closed triangles* β-glucan. Values are means + SD of three independent experiments. **e**, **f** Rates of ^14^C incorporation (dpm cell^−1^ h^−1^) into the various fraction in LP and SP cells, respectively. For time courses of total ^14^C fixation in LP and SP cells see Fig. 2. Values are means + SD

After fractionation of ^14^C-labeled photosynthetic products according to the method established in this study (Fig. 1), ^14^C radioactivity in each fraction was determined separately. ^14^C incorporation into all fractions increased linearly in both LP and SP cells, although the rates were higher in LP than SP cells (Fig. 3). ^14^C was mostly incorporated into the LMC-dominant fraction (fraction B), with ca. 40 and 45 % of total ^14^C fixation in LP and SP cells, respectively (Fig. 3c, d). According to the fractionation data, in which 24-h ^14^C-labeled cells were used as test materials (Supplementary Table S1), ^14^C-LMC occupied ca. 75 % of ^14^C in the LMC-dominant fraction (Supplemental Fig. S2). Finally, we calculated that ^14^C-LMC accounted for ca. 35 and 33 % of total ^14^C fixation in LP and SP cells, respectively (plotted as a plus (+) in Fig. 3c, d).

Lipids other than alkenones were the second most dominant products, containing 25–30 % of the total ^14^C-fixed in LP cells. This lipid fraction is expected to contain mainly membrane lipids and photosynthetic pigments. However, it contains no or only minor amounts of triacylglycerol (TAG), which is the major storage lipid in most microalgae, as reported by Volkman et al. (1986). We did not perform further analysis of ^14^C-polar lipids since these polar lipids are components of membranes. The composition of polar lipids in *E. huxleyi* has been reported previously (Bell and Pond 1996). The third most dominant products were alkenones and AP*ext*, which each represented ca. 18 % of total ^14^C fixation in LP cells (Fig. 3a, c). The percentage ^14^C incorporation into whole lipids other than alkenones and AP*ext* was almost identical, ca. 20 % of total ^14^C fixation in SP cells (Fig. 3c, d). Little ^14^C-labeled AP*int* or β-glucan was produced by either LP or SP cells (Fig. 3).

### TLC Analysis of the ^14^C-LMC-dominant Fraction

**Fig. 4:**
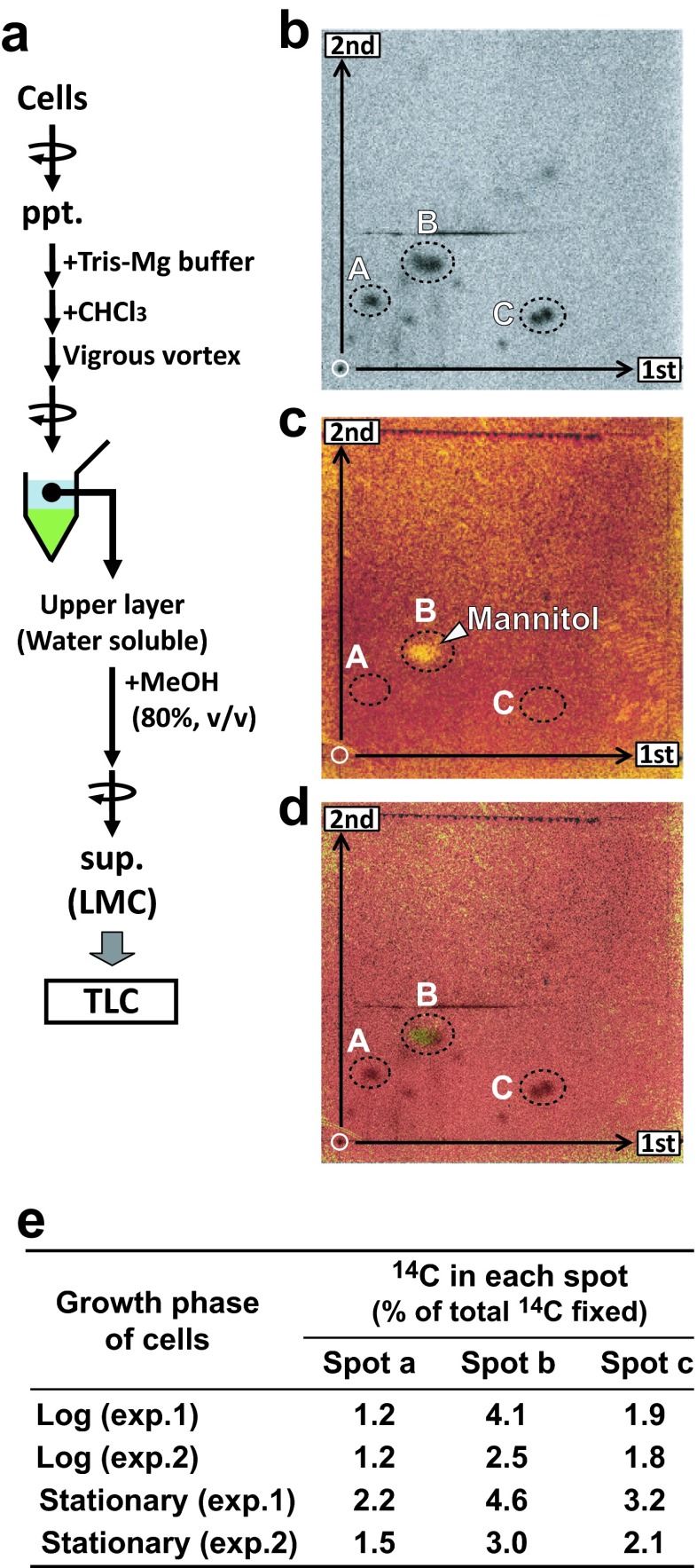
Two-dimensional TLC analysis of ^14^C-LMC produced by the coccolithophorid *E. huxleyi* NIES 837 under continuous illumination for 24 h. **a** Fractionation protocol for the preparation of LMC for TLC analysis. **b** Autoradiogram. **c** Mannitol spot (shown by *arrow*) visualized by KMnO_4_ reagent. **d** A merged image of the autoradiogram (**b**) and chemical staining of mannitol (**c**). The TLC images presented here are representative of two independent experiments using LP cells. **e** Quantification of ^14^C-mannitol (expressed as % of total ^14^C fixation). Results of two independent experiments (exp. 1 and 2) are shown

Obata et al. (2013) suggested that mannitol is used for carbon storage in *E. huxleyi.* To estimate the contribution of mannitol to the LMC-dominant fraction, we analyzed the molecular composition of ^14^C-LMC by thin-layer chromatography. However, detailed analysis of ^14^C-LMC was difficult since LMC is trapped by the gel in the spin column in the new fractionation method (Fig. 1). Therefore, we used another fractionation method to analyze ^14^C-LMC, namely, extraction using the water/chloroform two-phase partitioning method followed by 80 % methanol extraction (Fig. 4a). To identify the mannitol spot, commercial standard mannitol was added to ^14^C-LMC samples for simultaneous detection of ^14^C-spots and mannitol spots on the same TLC plate (Fig. 4b–d) (see the Materials and Methods). Three major ^14^C-spots appeared on the autoradiogram (spot A–C) and mannitol was detected as spot b (Fig. 4b–d). ^14^C in all spots was less than 5 % of total ^14^C-fixation (Fig. 4e). However, as the mannitol spot appeared to be multiple spots, further detailed quantitative analysis is necessary for high-quality estimation.

### Analysis of ^14^C-lipids

Lipids were separated into a chloroform-soluble fraction; i.e., the lower layer in the chloroform/water two-phase partitioning method (Fig. 1). When the lipid fraction was analyzed by TLC, two major radioactive spots in bands I and II were identified (Supplementary Fig. S1). Using gas chromatography (GC) analysis, bands I and II were identified as C_38_ and C_37_ alkenones, respectively (Supplementary Fig. S1b). C_39_ alkenones were not detected in the two major bands, possibly due to the low C_39_ alkenone production by strain NIES837 grown at 20 °C, as reported previously (Ono et al. 2009). The sum of C_37_ and C_38_ alkenones identified and quantified using TLC was expressed as “alkenones” in the present study. The alkenones accounted for ca. 17 % of total ^14^C-fixation in the 24-h ^14^C-labeling experiment in both LP and SP cells, while the ^14^C ratios in lipids other than alkenones were 25 and 18 % in LP and SP cells, respectively (Fig. 3c, d). Although we did not quantify the amount of TAG, there were no major bands of TAG in our TLC analysis, indicating no TAG qualitatively. These results are same as that in previous reports (Volkman et al. 1986; Eltgroth et al. 2005).

### Analysis of the ^14^C-polysaccharide Fraction

The polysaccharide fraction was divided into three subfractions, AP*ext* and AP*int* for acid polysaccharides and β-glucan as a neutral polysaccharide (Fig. 1). ^14^C-β-glucan comprised only 0.8 and 0.4 % of total ^14^C fixation in LP and SP cells, respectively (Fig. 3), despite its being assumed previously to be a storage compound in *E. huxleyi*. ^14^C-AP*int*, presumably located in the coccolith vesicles, comprised 1.3 and 2.5 % of total ^14^C fixation in LP and SP cells, respectively, whereas ^14^C-AP*ext*, synthesized intracellularly and then excreted to the cell surface with coccoliths, comprised 13 and 18 % of total ^14^C fixation in LP and SP cells, respectively (Fig. 3c, d). Total ^14^C-AP (AP*int* + AP*ext*) comprised 14 and 20 % of total ^14^C fixation in LP and SP cells, respectively.

### Catabolism of ^14^C-Labeled Compounds in the Dark in Light/Dark Transient Experiments

**Fig. 5:**
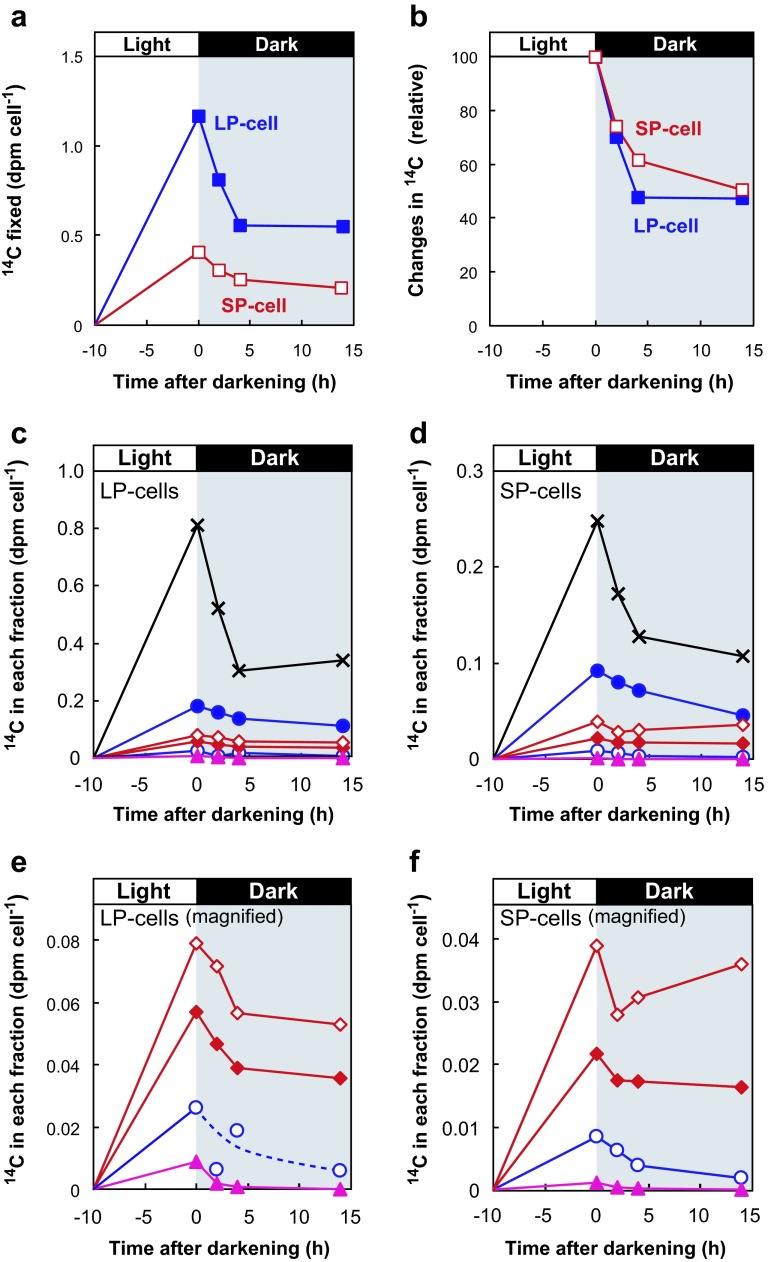
Changes in ^14^C in various fractions during light/dark transition in the coccolithophore *E. huxleyi* NIES837. First, cells were photosynthetically labeled with ^14^C using NaH^14^CO_3_ as the substrate for 10 h, and then transferred to dark conditions. **a** Changes in ^14^C incorporation into LP cells (*closed squares*) and SP cells (*open squares*) expressed as actual radioactivity (dpm cell^−1^). **b** Relative amount of ^14^C under dark conditions (% change) in LP (*closed squares*) and SP cells (*open squares*), respectively. **c**, **d** Changes in ^14^C incorporation into each metabolite in the LMC-dominant fraction (crosses), AP*ext* (closed circles), lipids other than alkenones (*open diamonds*), alkenones (*closed diamonds*), AP*int* (*open circles*), and β-glucan (*closed triangles*) expressed as actual activity (dpm cell^−1^) in LP and SP cells, respectively. **e**, **f** Graphs with magnified *y*-axis of Fig. 5c, d for lipids other than alkenones (*open diamonds*), alkenones (*closed diamonds*), AP*int* (*open circles*), and β-glucan (*closed triangles*) in LP and SP cells, respectively. Representative results of two independent experiments are shown

To evaluate utilization of photosynthetic ^14^C-products in the dark, *E. huxleyi* cells were transferred from light to dark conditions. After transition to dark conditions, total ^14^C in cells decreased rapidly and reached a steady level after a 4-h incubation in the dark in both LP and SP cells (Fig. 5a, b). Immediately following the transfer of cells from light to dark conditions, the ^14^C-LMC-dominant fraction, of which mannitol comprises ca. 16 % corresponding to 5 % of total ^14^C-fixation, decreased rapidly. ^14^C-alkenones decreased to ca. 70 % of the initial level during the first 4 h of incubation in the dark. These trends were similar in both LP and SP cells (Fig. 5c, d).

**Fig. 6:**
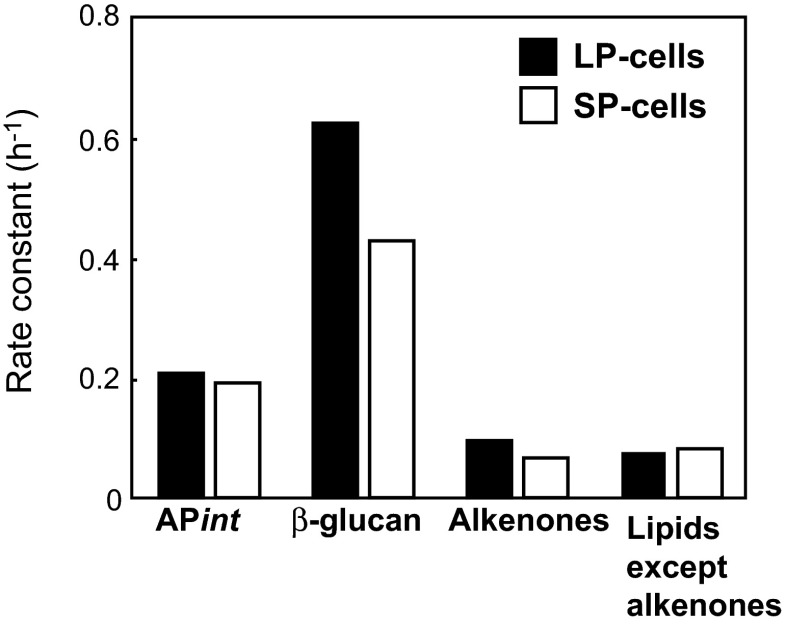
Rate constant of the decrease in ^14^C in intracellular macromolecules during a 4-h dark period in LP (*solid bars*) and SP cells (*open bars*). To calculate the rate constant, ^14^C decrease in each fraction was fit to exponential curves using the following equation: *R*_(*t*)_ = *R*_0_ *e* ^−*kt*^. In this equation, *R*_0_ and *R*_(*t*)_ are ^14^C-radioactivities in each fraction (dpm (10^3^ cells)^−1^) at time zero and *t*, respectively*. k* rate constant. Data in Fig. 5c, d were used for calculations

Since most ^14^C-labeled major fractions decreased exponentially during the first 4 h of incubation under dark conditions, ^14^C decreases of intracellular macromolecules were fit to exponential curves to calculate the rate constant using the following equation: *R* (t) = *R*_0_*e*^−*kt*^ (Fig. 6; Supplemental Fig. S3). In this equation, *R*_0_ and *R* (*t*) represent the radioactivity of ^14^C in each fraction (dpm cell^−1^) at time 0 and *t*, respectively. *k* is the rate constant of the degradation reaction. The rate constant of β-glucan was considerably higher than that of other intracellular macromolecules in both LP and SP cells (Fig. 6). These results showed a marked difference between β-glucan and alkenones as storage compounds, namely, alkenones function as a slowly degradable large carbon pool while β-glucan functions as a rapidly degradable small carbon pool. β-glucan cannot be considered a major carbon/energy storage compound since it is present at extremely low concentrations (Figs. 3 and 5e, f). The actual calculated concentrations of ^14^C released from alkenones during the first 4 h of incubation under dark conditions were two- and fivefold greater than that of β-glucan in LP and SP cells, respectively (Fig. 5e, f).

## Discussion

### Low Carbon Flux into β-glucan Is a Feature of Carbon Metabolism in *E. huxleyi*

Carbon metabolism in the haptophyte alga *E. huxleyi*, a secondary endosymbiotic alga, is distinguished from that in primary endosymbiotic algae by the production of unique photosynthetic products, such as alkenones, AP, and β-glucan. Here, we revealed that 17 % of fixed ^14^C is distributed to alkenones, while ^14^C in β-glucan was less than 1 % in both LP and SP cells (Fig. 3). This result demonstrated that fixed carbons are stored mainly as C_37_ and C_38_ alkenones, not as β-glucan. Another potential carbon storage resource is the LMC fraction, which accumulated ca. 30 % of cellular ^14^C, but contains various components (Fig. 3).

Generally, carbohydrates such as α/β-glucans are known to be major storage compounds stored during photosynthesis and preferentially utilized as primary energy/carbon sources in the dark in many phytoplankton species, while lipids play a role in slow, degradable carbon storage (Ricketts 1966; Handa 1969). However, our results demonstrate that β-glucan is not a major energy/carbon storage in *E. huxleyi* since ^14^C-flux into β-glucan for storage was extremely low, and therefore the amount of degradation was also very low (Fig. 5; Supplemental Fig. S3).

In diatoms, which are known to have evolved through secondary endosymbiosis of red alga, both synthesis and accumulation of β-glucan was stimulated under nutrient limited conditions, especially N-limitation (Myklestad 1989). In a diatom *Skeletonema costatum*, ca. 30 % of fixed ^14^C was incorporated into β-glucan in LP cells, and the value was higher in SP cells (Myklestad 1989). These trends are opposite to those observed in the coccolithophore *E. huxleyi*.

We previously reported that availability of inorganic phosphate (Pi) regulates carbon flux between β-glucan and AP in *E. huxleyi* (Kayano and Shiraiwa 2009). When Pi is sufficient, β-glucan synthesis is stimulated while AP synthesis is suppressed (Kayano and Shiraiwa 2009). This tendency was also observed in the present study; ^14^C in β-glucan of LP cells was twofold that in SP cells (Fig. 3c, d; 0.8 % in LP cells and 0.4 % in SP cells). According to these results, *E. huxleyi* synthesizes β-glucan when nutrients are sufficient. Although β-glucan is rapidly produced even in *E. hyxleyi* LP cells, the amount of ^14^C flux into β-glucan was much lower than that in diatoms. This result suggested that low carbon flux into β-glucan is a potent physiological property of *E. huxleyi.* However, it remains possible that β-glucan synthesis becomes dominant under specific conditions.

We demonstrated that β-glucan is not the major carbon/energy storage molecule in *E. huxleyi*. These features are not common in all coccolithophorids since the non-alkenone-producing coccolithophore, *Pleurochrysis haptonemofera*, produces β-glucan as the major carbon storage molecule (Hirokawa et al. 2008). The present study observed differences in the function of β-glucan between alkenone-producing and non-alkenone-producing coccolithophores.

### Alkenones Function in Storage in *E. huxleyi*

^14^C flux into alkenones were ca. 17 % in both LP and SP cells, demonstrating that alkenones are the major carbon/energy storage macromolecule in *E. huxleyi*. Alkenones were constantly synthesized through cell growth, suggesting that alkenone synthesis is not regulated by nutrient conditions. This result is consistent with previous studies showing the constant ratio of alkenones to total organic carbon in LP and SP cells of *E. huxleyi* (Conte et al. 1998). We showed constant biosynthesis of alkenones (Fig. 3), while coccolith production is enhanced by Pi-limitation (Kayano and Shiraiwa 2009). Therefore, alkenones are unlikely to function as a buoyancy regulator to compensate for the heaviness of coccoliths, since coccolith biosynthesis and alkenone biosynthesis are regulated independently.

In the natural environment, *E. huxleyi* may be exposed to dark periods of various lengths depending on their ecosystem conditions. By means of a dark incubation experiment that evaluated the alkenone degradation rate (Fig. 5), we demonstrated that alkenones are actively metabolized and can function as a carbon/energy source during the daily light/dark cycle. In addition to our data on short-term experiments (Fig. 5, several hours of dark period), the degradation of alkenones was observed in darkness after several days (Epstein et al. 2001; Prahl et al. 2003; Eltgroth et al. 2005; Pan and Sun 2011) and 25 % of alkenones remained after 5 days of darkness (Prahl et al. 2003). Considering the global distribution of *E. huxleyi*, alkenones may play key roles as important carbon storage molecules during circulation-mediated vertical transport of algal cells to 100–200 m depths.

Fernández et al. (1996) reported the reallocation of ^14^C from total lipids to proteins under dark conditions, although alkenones were not examined independently. It is possible that stored alkenones are used not only for energy but also serve as a carbon source for protein synthesis under dark conditions in *E. huxleyi*. Patterns of ^14^C incorporation into lipids other than alkenones were similar to those for alkenones, especially in LP cells (Fig. 5e). Similarly, lipids other than alkenones, including polar lipids and pigments, which are components of membranes, may function in carbon and energy storage.

What is the advantage of accumulating alkenones as storage compounds? Triacylglycerol (TAG) is a general storage lipid in various microalgae (Yoshida et al. 2012), but *E. huxleyi* produces only trace amounts of TAG (Volkman et al. 1986). In comparison with TAG, the unique features of alkenones are their extremely long carbon chain lengths (C_37_–C_39_), the *keto*-group, and *trans*-double bonds. Rontani et al. (1997) showed that *trans*-unsaturated alkenones are more resistant to photochemical degradation than other lipids with *cis*-unsaturated bonds. *E. huxleyi* frequently produces blooms at the ocean surface where it is exposed to very high irradiance (Tyrrell and Merico 2004); the high resistance of alkenones to photochemical degradation may be advantageous under such conditions (Rontani et al. 1997). *E. huxleyi* is exposed to various environmental conditions from high irradiance to prolonged dark periods according to climate change and vertical mixing. The photostability of alkenones is likely important for this organism to adapt to sudden changes in the light environment. However, further direct analysis is required to support this hypothesis.

### Mannitol and LMC Function as Water-Soluble Carbon Storage Molecules in *E. huxleyi*

Recently, Obata et al. (2013) suggested that some LMCs, such as mannitol, could act as storage compounds in *E. huxleyi*. In the present study, almost 40 % of total ^14^C-fixed was incorporated into the LMC-dominant fraction (Fig. 3). ^14^C-mannitol was estimated to comprise less than 5 % of total ^14^C-fixed, which is ca. 30 % of the ^14^C in alkenones. Obata et al. (2013) analyzed ^13^C flux into LMC including mannitol, but no data on macromolecules are available. On the other hand, our analysis provided quantitative data on ^14^C flux into mannitol and alkenones and revealed that ^14^C flux into alkenones was higher than that into mannitol by comparing the production profile of both metabolites (Fig. 3). The ^14^C incorporation into the LMC-dominant fraction was not saturated within the 24-h labeling period (Fig. 3), suggesting that LMC has a large capacity for carbon storage. We consider that not only mannitol but also the whole pool of LMC may function in carbon storage in combination with alkenones. Linear ^14^C incorporation into the LMC fraction in the marine brown alga *Eisenia bicyclis*, which accumulates high amounts of mannitol as a storage compound, has been reported (Yamaguchi et al. 1966). These trends differ from other reports that ^14^C incorporation into photosynthetic primary intermediates generally attains a steady level on the order of minutes to an hour, as in the freshwater cryptophyte *Chroomonas* sp. (Suzuki and Ikawa 1985) and freshwater chlorophyte *Chlorella vulgaris* (Miyachi et al. 1978; Nakamura and Miyachi 1982). The biosynthetic pathway for mannitol in brown algae was revealed as photosynthetic synthesis via C_3_-intermediates produced in the Calvin–Benson cycle, hexose phosphate, and mannitol-1-phosphate (Yamaguchi et al. 1969).

In the present study, we showed high ^14^C-flux into LMC in the light and a rapid decrease in ^14^C-LMC in the dark. However, further studies are required to identify other LMC and their metabolic profiles to clarify how mannitol and other LMCs function in carbon storage. Mannitol has various functions, including as a storage compound, compatible solute, energy source to regenerate reducing power, and agent for scavenging reactive oxygen species, in various algae (Iwamoto and Shiraiwa 2005). In addition to mannitol, several other major unidentified ^14^C compound spots were found by TLC (Fig. 4b). These compounds may include dimethylsulfoniopropionate (DMSP), as *E. huxleyi* is known to synthesize DMSP as a compatible solute (Franklin et al. 2010). Although changes in salinity are minimal in the ocean, compatible solutes should be synthesized according to the increase in cell volume or cell number. One of the ^14^C-LMC spots (Fig. 5b) is expected to be DMSP, which is known to be a compatible solute of *E. huxleyi* (Franklin et al. 2010).

### Acid Polysaccharides Are Major Products Associated with Coccoliths in *E. huxleyi*

^14^C incorporation into AP*ext* was 13–18 % of total ^14^C-fixed, which is comparable with that into alkenones (Fig. 3). AP*ext* was also decreased under dark conditions (Fig. 5). However, AP*ext* is unlikely to be a storage polysaccharide since it is embedded in or covering the coccoliths on the cell surface and so is a structural component rather than a storage compound (Van Emburg et al. 1986). A proportion of AP*ext* is released into the medium, where it is degraded by bacterial activity (Van Oostende et al. 2013). Since the *E. huxleyi* strain used in this study (NIES837) is not from a completely axenic culture, it is possible that the decrease in ^14^C-AP*ext* under dark conditions may be due to the release of AP into the medium and subsequent degradation by bacteria.

In conclusion, we observed high ^14^C-flux into lipids including alkenones and LMCs, while ^14^C-flux into β-glucan was low in *E. huxleyi*. ^14^C-flux into alkenones was constant in both LP and SP cells, suggesting that alkenone biosynthesis was not regulated by nutrients. Coccolithophores and other secondary endosymbiotic algae, such as diatoms, are proposed to have distinct enzyme sets and regulatory mechanisms for central carbon metabolism (Hockin et al. 2012). Studies on the unique carbon metabolism of secondary endosymbiotic algae will provide new insight onto the diversity and evolution of photosynthetic carbon metabolism in eukaryotes. According to biogeochemical evidence, the coccolithophore *E. huxleyi* may be a source of petroleum since long-chain hydrocarbons are present in crude oil. The high concentration of alkenones in this organism supports such biogeochemical evidence as alkenones are known to have high potential for crude oil production in comparison with other microalgae (Yamane et al. 2013).

## Electronic Supplementary Material

Supplementary Fig. S1 (PPTX 224 kb)

Supplementary Fig. S2 (PPTX 124 kb)

Supplementary Fig. S3 (PPTX 94 kb)

Supplementary Table S1 (PPTX 52 kb)
