# Supplementary material for: Quantitative Analysis of Carbon Flow into Photosynthetic Products Functioning as Carbon Storage in the Marine Coccolithophore, Emiliania huxleyi
Source: Mar Biotechnol (NY). 2015 Apr 15;17(4):428–40. doi: 10.1007/s10126-015-9632-1 (PMC4486895; doi:10.1007/s10126-015-9632-1)
Supplement: Supplementary file 1 — (PPTX 224 kb) [file 10126_2015_9632_MOESM1_ESM.pptx]

## Slide 1
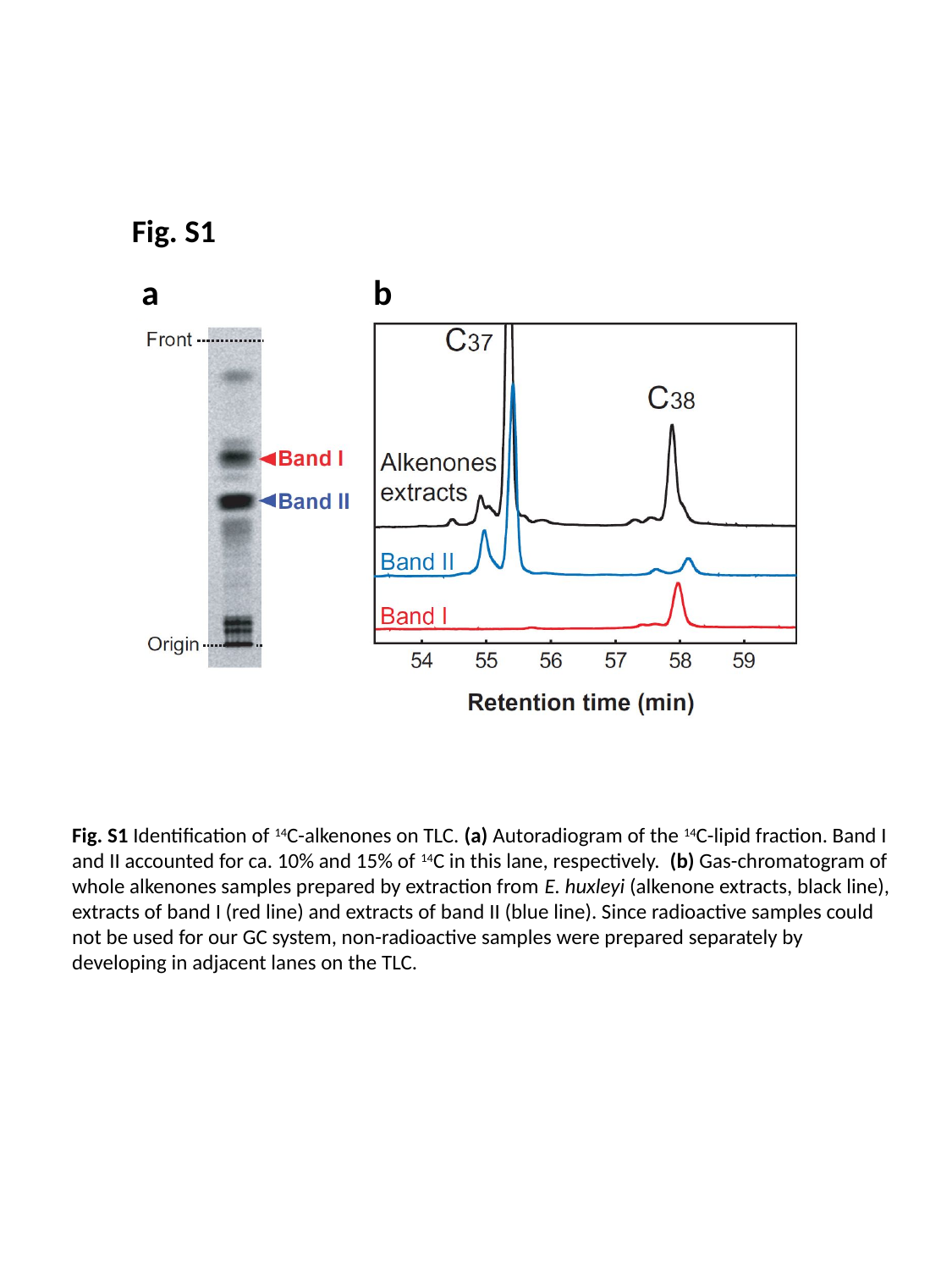

Fig. S1
a
b
Fig. S1 Identification of 14C-alkenones on TLC. (a) Autoradiogram of the 14C-lipid fraction. Band I and II accounted for ca. 10% and 15% of 14C in this lane, respectively. (b) Gas-chromatogram of whole alkenones samples prepared by extraction from E. huxleyi (alkenone extracts, black line), extracts of band I (red line) and extracts of band II (blue line). Since radioactive samples could not be used for our GC system, non-radioactive samples were prepared separately by developing in adjacent lanes on the TLC.
