# Supplementary material for: Quantitative Analysis of Carbon Flow into Photosynthetic Products Functioning as Carbon Storage in the Marine Coccolithophore, Emiliania huxleyi
Source: Mar Biotechnol (NY). 2015 Apr 15;17(4):428–40. doi: 10.1007/s10126-015-9632-1 (PMC4486895; doi:10.1007/s10126-015-9632-1)
Supplement: Supplementary file 2 — (PPTX 124 kb) [file 10126_2015_9632_MOESM2_ESM.pptx]

## Slide 1
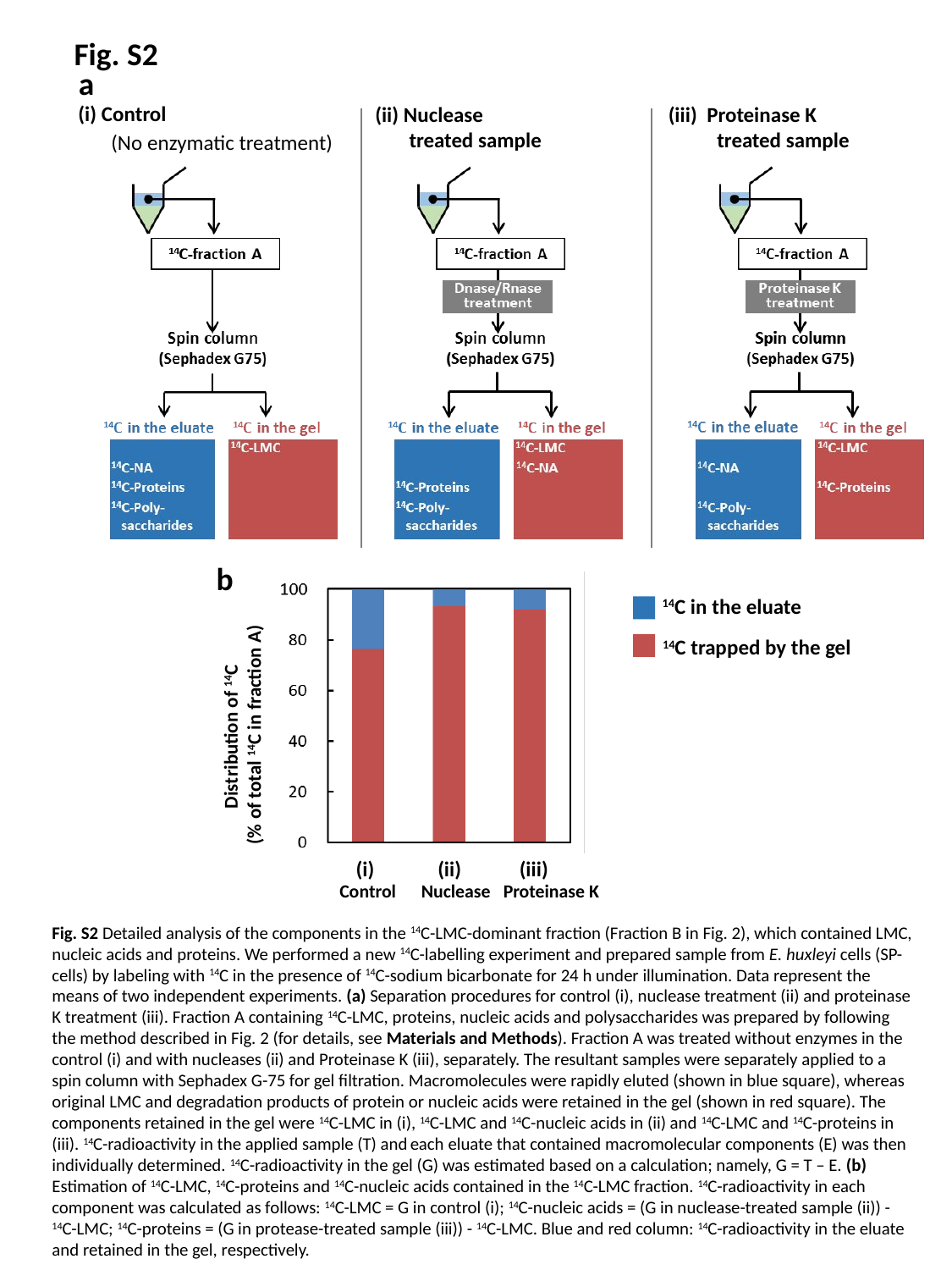

Fig. S2
a
(i) Control
 (No enzymatic treatment)
(ii) Nuclease
 treated sample
(iii) Proteinase K
 treated sample
b
14C in the eluate
14C trapped by the gel
Distribution of 14C
(% of total 14C in fraction A)
(i)
(ii)
(iii)
Control
Nuclease
Proteinase K
Fig. S2 Detailed analysis of the components in the 14C-LMC-dominant fraction (Fraction B in Fig. 2), which contained LMC, nucleic acids and proteins. We performed a new 14C-labelling experiment and prepared sample from E. huxleyi cells (SP-cells) by labeling with 14C in the presence of 14C-sodium bicarbonate for 24 h under illumination. Data represent the means of two independent experiments. (a) Separation procedures for control (i), nuclease treatment (ii) and proteinase K treatment (iii). Fraction A containing 14C-LMC, proteins, nucleic acids and polysaccharides was prepared by following the method described in Fig. 2 (for details, see Materials and Methods). Fraction A was treated without enzymes in the control (i) and with nucleases (ii) and Proteinase K (iii), separately. The resultant samples were separately applied to a spin column with Sephadex G-75 for gel filtration. Macromolecules were rapidly eluted (shown in blue square), whereas original LMC and degradation products of protein or nucleic acids were retained in the gel (shown in red square). The components retained in the gel were 14C-LMC in (i), 14C-LMC and 14C-nucleic acids in (ii) and 14C-LMC and 14C-proteins in (iii). 14C-radioactivity in the applied sample (T) and each eluate that contained macromolecular components (E) was then individually determined. 14C-radioactivity in the gel (G) was estimated based on a calculation; namely, G = T – E. (b) Estimation of 14C-LMC, 14C-proteins and 14C-nucleic acids contained in the 14C-LMC fraction. 14C-radioactivity in each component was calculated as follows: 14C-LMC = G in control (i); 14C-nucleic acids = (G in nuclease-treated sample (ii)) - 14C-LMC; 14C-proteins = (G in protease-treated sample (iii)) - 14C-LMC. Blue and red column: 14C-radioactivity in the eluate and retained in the gel, respectively.
