# Supplementary material for: Quantitative Analysis of Carbon Flow into Photosynthetic Products Functioning as Carbon Storage in the Marine Coccolithophore, Emiliania huxleyi
Source: Mar Biotechnol (NY). 2015 Apr 15;17(4):428–40. doi: 10.1007/s10126-015-9632-1 (PMC4486895; doi:10.1007/s10126-015-9632-1)
Supplement: Supplementary file 3 — (PPTX 94 kb) [file 10126_2015_9632_MOESM3_ESM.pptx]

## Slide 1
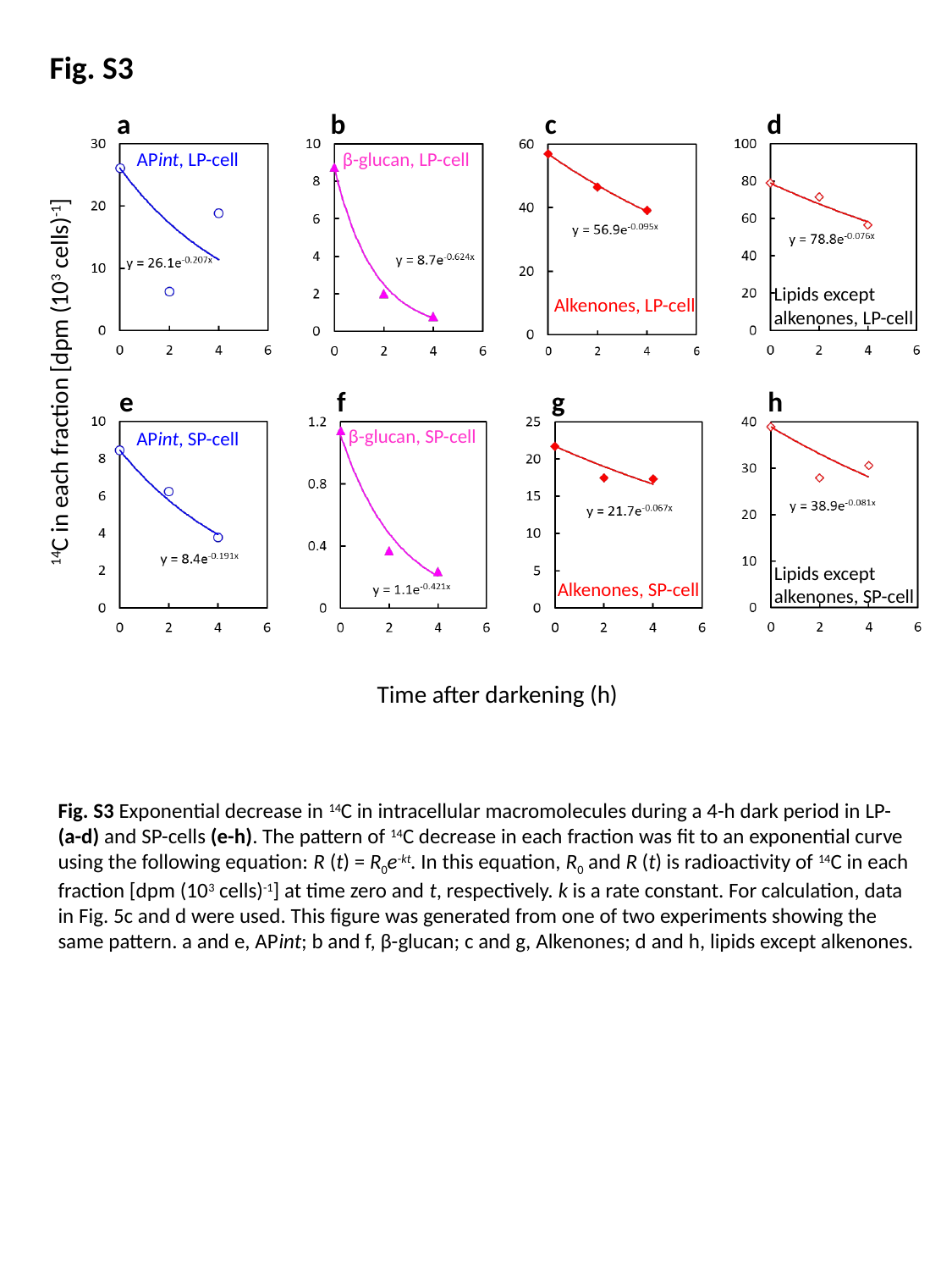

Fig. S3
a
b
c
d
APint, LP-cell
β-glucan, LP-cell
Lipids except
alkenones, LP-cell
Alkenones, LP-cell
14C in each fraction [dpm (103 cells)-1]
e
f
g
h
β-glucan, SP-cell
APint, SP-cell
Lipids except
alkenones, SP-cell
Alkenones, SP-cell
Time after darkening (h)
Fig. S3 Exponential decrease in 14C in intracellular macromolecules during a 4-h dark period in LP- (a-d) and SP-cells (e-h). The pattern of 14C decrease in each fraction was fit to an exponential curve using the following equation: R (t) = R0e-kt. In this equation, R0 and R (t) is radioactivity of 14C in each fraction [dpm (103 cells)-1] at time zero and t, respectively. k is a rate constant. For calculation, data in Fig. 5c and d were used. This figure was generated from one of two experiments showing the same pattern. a and e, APint; b and f, β-glucan; c and g, Alkenones; d and h, lipids except alkenones.
