# Supplementary figures and images for: Quantitative Analysis of Carbon Flow into Photosynthetic Products Functioning as Carbon Storage in the Marine Coccolithophore, Emiliania huxleyi
Source: Mar Biotechnol (NY). 2015 Apr 15;17(4):428–40. doi: 10.1007/s10126-015-9632-1 (PMC4486895; doi:10.1007/s10126-015-9632-1)

## Slide 1
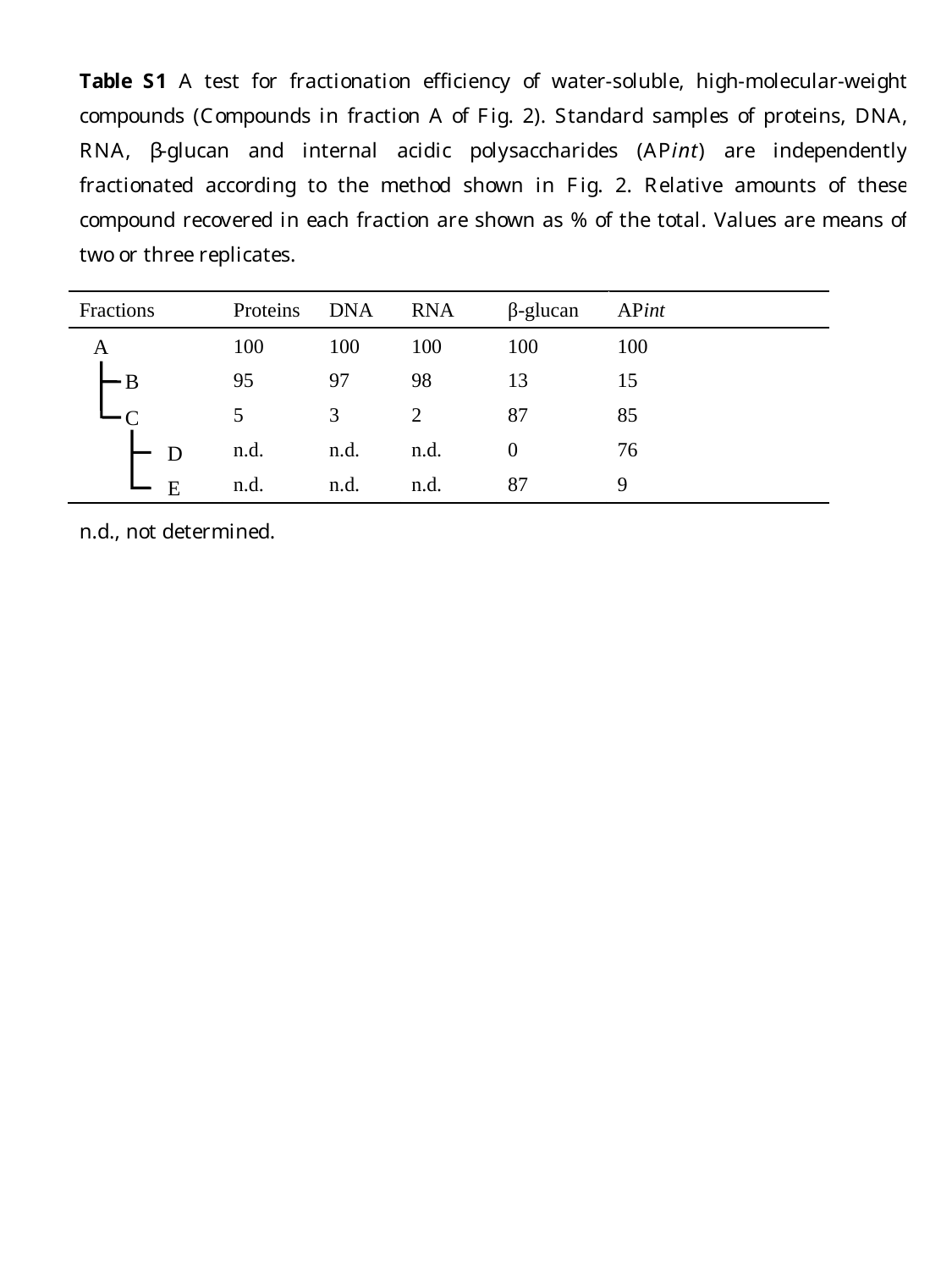

A
B
C
D
E

Supplement: Supplementary file 4 — (PPTX 52 kb) [file 10126_2015_9632_MOESM4_ESM.pptx]
